# Chronic Respiratory Symptoms and Lung Function in Agricultural Workers - Influence of Exposure Duration and Smoking

**DOI:** 10.3889/oamjms.2015.014

**Published:** 2015-01-13

**Authors:** Saso Stoleski, Jordan Minov, Dragan Mijakoski, Jovanka Karadzinska-Bislimovska

**Affiliations:** *Institute for Occupational Health of Republic of Macedonia - Skopje, WHO Collaborating Center, Ga2len Collaborating Center, Skopje, Republic of Macedonia*

**Keywords:** respiratory health, agriculture, spirometry, smoking, job exposure

## Abstract

**INTRODUCTION:**

Job exposure in agricultural workers often leads to respiratory impairment.

**AIM:**

To assess the influence of exposure duration and smoking on chronic respiratory symptoms and ventilatory capacity in agricultural workers.

**METHODS:**

A cross-sectional study covered 75 agricultural workers, compared with an equal number of office workers matched by age, exposure duration and smoking status. Standardized questionnaire was used to obtain data on chronic respiratory symptoms, job and smoking history. Lung functional testing was performed by spirometry.

**RESULTS:**

The prevalence of respiratory symptoms was higher in agricultural workers, with significant difference for cough (*P* = 0.034), and dyspnea (*P* = 0.028). Chronic respiratory symptoms among agricultural workers were significantly associated with duration of exposure (*P* < 0.05) and daily smoking (*P* < 0.01), as well as with daily smoking in controls (*P* < 0.01). The average values of spirometric parameters in exposed workers were significantly different for MEF_50_ (*P* = 0.002), MEF_75_ (*P* = 0.000), and MEF_25-75_ (*P* = 0.049). Obstructive changes in small airways in exposed workers were strongly related to exposure duration (*P* < 0.05) and smoking (*P* < 0.01). Agricultural workers with job exposure more than 15 years had more expressed adverse respiratory symptoms and lung function decline.

**CONCLUSION:**

The results confirmed the influence of agricultural exposure and daily smoking on chronic respiratory symptoms and airflow limitation, primarily targeting the small airways.

## Introduction

Respiratory diseases and lung functional impairment are worldwide well recognized occupational problems among agricultural workers. A vast number of epidemiological data obtained within last few decades indicate that they impose a higher morbidity and mortality from respiratory disorders compared to the general population or other occupational groups, even though there is a lower prevalence of smoking habit [1-3].

Pulmonary disorders among farmers may be caused by a wide variety of agents and hazards. The most frequent are organic dusts (grain, straw, hay) usually containing bacteria, moulds, mites and their excreta, as well as animal derivatives (dander, urine, feces). On the other hand, activities that embrace the soil (plowing, tilling, etc.) may expose farmers to inorganic silica dust. Agricultural work also includes some other type of hazards like chemical products (pesticides, fertilizers, paints, preservatives, and disinfectants), gases and fumes, but also biological agents [3-5].

Farmers are often exposed to high concentration of dust while tilling the soil and harvesting crops. Soil is the main source of this complex inorganic dust fraction. While the association between exposure to respirable silica dust and development of respiratory diseases is well known, further research interest is focused on the pathologic potential of other soil silicates [6].

Subjects that are engaged in agriculture may be potentially exposed to a very wide range of respiratory hazards including inorganic soil dust, organic dust rich in microorganisms, mycotoxins or allergens, decomposition gases, pesticides etc. Most of these exposures occur when exposed workers have animal contact, in the process of harvesting, processing or storing grains and other plants, or while treating the soil, plants, or stables with pesticides and disinfectants [7].

Already mentioned and explained substances and hazards present in farmers’ environment are known to cause organic dust syndrome, chronic bronchitis, allergic and non-allergic asthma, asthma-like syndrome, chemical and hypersensitivity pneumonitis [8-11], allergic and non-allergic rhinitis [12]. Chronic respiratory symptoms are common in agricultural workers, and they mostly depend on the type of farming practice [9]. Exposures can cause disease of either the upper or the lower respiratory tract, or both.

Nevertheless, the most frequent, but also well studied respiratory diseases associated with farming are allergic rhinitis. The same individual can, and usually is affected simultaneously, and the most common causes of these two pathological entities are storage mites, urinary proteins, and cow dander [13, 14].

Chronic bronchitis and chronic obstructive pulmonary disease (COPD) should be regarded as occupational risks in farming by many epidemiological studies performed in many countries [15, 16]. Dusts, gases and fumes inhalation may often lead to respiratory irritation and chronic inflammation, while prolonged exposure can provoke bronchial obstruction, but also a loss of lung parenchyma elasticity [17].

Higher prevalence of respiratory diseases, such as asthma, chronic bronchitis, extrinsic allergic alveolitis, organic dust toxic syndrome, and interstitial lung disorders is registered among subjects with workplace exposure to organic dust. Sometimes, there are complex respiratory pathologies, which can lead to a presentation that is difficult to recognize, with a mixture of respiratory tract irritation and/or inflammation signs [18].

Chronic airflow limitation may result from airway obstruction, but also by the loss of elastic recoil in the parenchyma [19]. The primary early stage pathologic component is an inflammatory response within the peripheral airways. Agricultural exposures suggested as potential initiators of the airway inflammatory process, include dusts from cereal grains, animal feed and soils, gases and fumes, as well as microorganisms or their components, such as endotoxins and fungi [20].

Generally, smoking in farmers is lower compared to persons in most other occupations [21], and this tendency is demonstrated by the results of general health surveys, cancer case-control studies, and studies of respiratory disease among farmers and rural populations.

In the present study we have compared the prevalence of chronic respiratory symptoms and spirometric parameters, but also its relation to duration of exposure and smoking between agricultural workers and office workers, which were matched by gender and age.

## Subjects and Methods

### Study design and setting

The research team carried out a cross-sectional survey in the Center for Respiratory Functional Diagnostics at the Institute for Occupational Health of R. Macedonia, Skopje - WHO Collaborating Center for Occupational Health and GA^2^LEN Collaborating Center in the period April 2013 and September 2014.

### Subjects

We have examined 75 subjects, 44 males and 31 females aged 21 to 64 years working as agricultural workers with duration of employment 2 to 44 years (mean duration 21.4±5.2).

The agricultural workers participating in the survey were involved in crop farming and exposed to: dust, inappropriate climate, chemical agents, contact with plants, heavy manual work, loading, unfavorable body postures, repetitive hand movements, and work with sharp tools and devices. Their main agricultural activities were cultivating crops and vegetables, planting, digging in the fields, use of mechanized equipment, irrigation, and pesticide spraying.

For the study purposes depending on the exposure duration the examined subjects were divided in two subgroups: exposed less or more than 15 years.

Additionally, a very similar group of 75 office workers (43 males and 32 females) matched to agricultural workers by age, gender, duration of employment, and daily smoking was studied as a control.

If there was a chronic respiratory disease diagnosed by physician, the subjects were excluded from the study. All study subjects were informed about the study and gave their written consent for participation.

### Questionnaire

During the study, every subject was interviewed by a physician, also responsible for completion of the questionnaire. The standardized questionnaire covered questions about work history, chronic respiratory symptoms in the last 12 months, but also about the subjects’ smoking status.

Questions were asked about previous and current job, daily working time, job description, working conditions, job activities performed, and regular use of protective measures and equipment.

European Community for Coal and Steel questionnaire (ECCS-87), and the European Community Respiratory Health Survey (ECRHS) questionnaire were used to document chronic respiratory symptoms in the last 12 months (cough, phlegm, dyspnea, wheezing, and chest tightness) [22, 23].

The questionnaire also comprised a detailed smoking history, accompanying disease, and medication use. Classification of smoking status was done according to the World Health Organization (WHO) guidelines on definitions of smoking status [24].

Daily smoker was defined as a subject who smoked at the time of the survey at least once a day, except on days of religious fasting. Among daily smokers, we have evaluated lifetime cigarette smoking and daily mean of cigarettes smoked. Pack-years smoked (one pack-year denotes one year of smoking 20 cigarettes per day) were calculated according to the actual recommendations [25].

Ex-smoker was defined as a formerly daily smoker, no longer smokes. Passive smoking or exposure to environmental tobacco smoke (ETS) was defined as the exposure of a person to tobacco combustion products from smoking by others [26].

### Spirometry

Spirometry was performed in all subjects using spirometer Ganshorn SanoScope LF8 (Ganshorn Medizin Electronic GmbH, Germany) measuring the forced vital capacity (FVC), forced expiratory volume in one second (FEV_1_), FEV_1_/FVC ratio, and maximal expiratory flow at 50%, 75%, and 25-75% of FVC (MEF_50_, MEF_75_, and MEF_25-75_, respectively). The best result from three measurements of the values of FEV_1_ was recorded within 5% of each other. The spirometry results of were expressed as percentages of the predicted values according to the European Community for Coal and Steel (ECCS) norms [27].

### Statistical analysis

Statistica for Windows version 7 and Epi info 6 were used for data description and analysis. Continuous variables were expressed as mean values with standard deviation and categorical variables as numbers and percentages. The chi-square test (or Fisher’s exact test where appropriate) was used for testing differences in the prevalence of respiratory symptoms, while independent-samples T-test was applied for comparison of spirometric measurements. A *P*-value of less than 0.05 was considered statistically significant. Linear regression analysis was used to assess the independent effect of exposure duration in agriculture, smoking and age on lung functional parameters.

## Results

Demographic characteristics of the study subjects were similar in both agricultural workers and office controls (Table 1).

**Table 1:** Demographics of the study subjects.

Variable	Agricultural workers (n = 75)	Office workers (n = 75)
		
M/F ratio	1.4	1.3
Age range (years)	21 - 64	22 - 63
Age (years)	51.4 ± 7.3	52.7 ± 7.6
BMI (kg/m^2^)	23.9 ± 3.4	24.2 ± 3.6
Duration of employment (years)	21.4 ± 5.2	20.7 ± 4.9
Duration of employment more than 15 years	35 (46.7%)	34 (45.3%)
Duration of employment less than 15 years	40 (53.3%)	41 (54.7%)
Daily smokers	20 (26.7%)	22 (29.3%)
Life-time smoking (years)	18.3 ± 5.1	19.1 ± 5.6
Cigarettes / day	15.3 ± 7.2	16.4 ± 6.9
Pack-years smoked	12.4 ± 4.3	12.7 ± 4.1
Daily smokers with less than 10 pack-years smoked	9 (12%)	10 (13.3%)
Ex-smokers	4 (5.3%)	5 (6.7%)
Passive smokers	12 (16%)	11 (14.7%)

Numerical data are expressed as mean value with standard deviation; frequencies as number and percentage of study subjects with certain variable. BMI: body mass index; kg: kilogram; m: meter.

Prevalence of respiratory symptoms in the last 12 months was higher in agricultural workers than in office workers with significant difference for cough and dyspnea (Table 2).

**Table 2:** Prevalence of respiratory symptoms in the last 12 months in examined groups.

Respiratory symptoms in the last 12 months	Agricultural workers (n = 75)	Office workers (n = 75)	*P*-value*
Any respiratory symptom	22 (29.3%)	16 (21.3%)	0.259
Cough	15 (20.0%)	6 (8.0%)	0.034
Phlegm	8 (10.7%)	4 (5.3%)	0.230
Dyspnea	9 (12.0%)	2 (2.7%)	0.028
Wheezing	8 (10.7%)	3 (4.0%)	0.210
Chest tightness	6 (8.0%)	4 (5.3%)	0.743

Data are expressed as number and percentage of study subjects with certain variable.

* Tested by chi-square test or Fisher’s exact test where appropriate.

Prevalence of respiratory symptoms in the last 12 months was higher in agricultural workers with workplace exposure more than 15 years than in those with workplace exposure less than 15 years having significant difference for overall respiratory symptoms and dyspnea (Table 3).

**Table 3:** Prevalence of respiratory symptoms in the last 12 months in agricultural workers with duration of workplace exposure more and less than 15 years.

Respiratory symptoms in the last 12 months	Exposed ≥ 16 years (n = 42)	Exposed ≤ 15 years (n = 33)	*P*-value*
Any respiratory symptom	17 (40.5%)	5 (15.2%)	0.016
Cough	11 (23.1%)	4 (7.3%)	0.130
Phlegm	5 (11.9%)	3 (9.1%)	0.499
Dyspnea	8 (19.1%)	1 (3.1%)	0.034
Wheezing	6 (15.4%)	2 (9.1%)	0.298
Chest tightness	4 (9.5%)	2 (6.1%)	0.688

Data are expressed as number and percentage of study subjects with certain variable.

* Tested by chi-square test or Fisher’s exact test where appropriate.

Association of respiratory symptoms in agricultural workers with duration of exposure (≤15 and ≥16 years), current smoking, and passive smoking in agricultural workers and controls is shown in Table 4. The association between chronic respiratory symptoms in exposed daily smokers, exposure duration and smoking experience was statistically significant (*P*<0.05), as well as between chronic respiratory symptoms and pack-years smoked (*P*<0.01). In unexposed controls, significance was found with daily smoking (*P*<0.01). A joint effect of the exposure duration, daily smoking, smoking experience, and pack-years smoked was significantly associated with chronic respiratory symptoms in agricultural workers. Concerning individual chronic respiratory symptoms, cough, wheezing, and dyspnea showed a significant association with duration of exposure. Chronic cough and phlegm in both groups, as well as dyspnea in agricultural workers, were significantly associated with daily smoking. There was not a significant association of overall or any individual chronic respiratory symptom with passive smoking in both groups.

**Table 4:** Association of respiratory symptoms with exposure duration and smoking habit.

Variable	Agricultural workers (n = 75)	*P*-value*	Office workers (n = 75)	*P*-valuee*
Workplace exposure ≥ 16 yrs with respiratory symptoms	17/42 (40.5%)	0.043	-	-
Workplace exposure ≤ 15 yrs with respiratory symptoms	5/33 (15.2%)			
Daily smokers with respiratory symptoms	12/22 (54.5%)	0.001	12/16 (75.0%)	0.001
Daily smokers without respiratory symptoms	8/53 (15.1%)		10/59 (16.9%)	
Passive smokers with respiratory symptoms	5/22 (22.7%)	0.318	4/16 (25.0%)	0.233
Passive smokers without respiratory symptoms	7/53 (13.2%)		7/59 (11.8%)	

Data are expressed as number and percentage of examinees with certain variable with and without chronic respiratory symptoms. Yrs: years.

* Tested by Chi-square test.

Mean values of spirometric parameters were lower in agricultural workers with statistical difference for mean values of MEF_50,_ MEF_75_, and MEF_25-75_ (Table 5).

**Table 5:** Mean values of spirometric parameters in examined groups.

Spirometric parameter	Agricultural workers (n = 75)	Office workers (n = 75)	*P*-value*
FVC (% pred)	84.2 ± 8.6	86.4 ± 8.9	0.126
FEV_1_ (% pred)	82.7 ± 8.3	85.1 ± 8.2	0.077
FEV_1_/FVC%	73.8 ± 4.1	74.3 ± 4.6	0.483
MEF_50_ (% pred)	56.4 ± 6.2	60.3 ± 6.4	0.002
MEF_75_ (% pred)	52.8 ± 5.7	60.5 ± 7.1	0.000
MEF_25-75_ (% pred)	61.3 ± 7.2	63.8 ± 8.2	0.049

Data are expressed as mean value with standard deviation. FVC: forced vital capacity; FEV_1_: forced expiratory volume in 1 second; MEF_50_, MEF_75_, MEF_25-75_: maximal expiratory flow at 50%, 75%, and 25-75% of FVC, respectively; % pred: % of predicted value.

* Tested by independent-sample *T*-test.

Concerning spirometric changes in agricultural workers and controls, significant difference between the groups was found for small airways obstruction.

Mean values of spirometric parameters were lower in agricultural workers exposed for more than 15 years than in those exposed less than 15 years with statistical significance for MEF_50_, MEF_75_ and MEF_25-75_ (Table 6).

**Table 6:** Mean values of spirometric parameters in agricultural workers with duration of workplace exposure more and less than 15 years.

Spirometric parameter	Exposed ≥ 16 years (n = 42)	Exposed ≤ 15 years (n = 33)	*P*-value*
FVC (% pred)	83.1 ± 8.4	84.7 ± 8.7	0.422
FEV_1_ (% pred)	80.3 ± 6.5	81.9 ± 7.4	0.322
FEV_1_/FVC%	72.1 ± 3.6	73.7 ± 4.2	0.080
MEF_50_ (% pred)	53.4 ± 5.1	55.9 ± 5.3	0.041
MEF_75_ (% pred)	49.4 ± 5.3	52.2 ± 5.6	0.029
MEF_25-75_ (%pred)	59.3 ± 6.3	61.8 ± 8.2	0.038

Data are expressed as mean value with standard deviation. FVC: forced vital capacity; FEV_1_: forced expiratory volume in 1 second; MEF_50_, MEF_75_, MEF_25-75_: maximal expiratory flow at 50%, 75%, and 25-75% of FVC, respectively; % pred: % of predicted value.

* Tested by independent-sample *T*-test.

The association of obstructive pattern in agricultural workers with duration of exposure, as well as daily smoking, smoking experience, and pack-years smoked in agricultural workers and office controls is shown in Table 7. The association between obstructive pattern and daily smoking, smoking experience, and pack-years smoked was not significant in both exposed and unexposed current smokers. A joint effect of duration of exposure, current smoking, smoking experience, and pack-years smoked on obstructive pattern development in agricultural workers was not significant.

**Table 7:** Association of obstructive pattern with exposure duration and smoking habit.

Variable	Agricultural workers (n = 75)	*P*-value*	Office workers (n = 75)	*P*-value*
Workplace exposure ≥ 16 yrs with obstructive pattern	9/42 (21.4%)	0.147	-	-
Workplace exposure ≤ 15 yrs with obstructive pattern	3/33 (9.1%)			
Daily smokers with obstructive pattern	5/12 (41.7%)	0.283	3/5 (60.0%)	0.118
Daily smokers without obstructive pattern	15/63 (23.8%)		19/70 (27.1%)	
Passive smokers with obstructive pattern	3/12 (25.0%)	0.394	2/5 (40.0%)	0.153
Passive smokers without obstructive pattern	9/63 (14.3%)		9/70 (12.9%)	

Data are expressed as number and percentage of examinees with certain variable with and without obstructive pattern. Yrs: years.

* Tested by Chi-square test.

The association of small airways obstructive changes in agricultural workers with duration of exposure, as well as daily smoking, smoking experience, and pack-years smoked in agricultural workers and office controls is shown in Table 8. The association between small airway obstructive changes in exposed daily smokers and smoking experience was statistically significant (*P*<0.01), as well as between small airways changes and exposure duration and pack-years smoked (*P*<0.05). The association between small airways obstructive changes and smoking experience, as well as pack-years smoked in unexposed smokers was not significant. A joint effect of exposure duration, daily smoking, smoking experience and pack-years smoked on small airways obstructive changes in agricultural workers was significant, but no significant association of small airway changes with passive smoking was found in both groups.

**Table 8:** Association of small airways obstructive changes with exposure duration and smoking habit.

Variable	Agricultural workers (n = 75)	*P*-value*	Office workers (n = 75)	*P*-value*
Workplace exposure ≥ 16 yrs with small airways obstructive changes	16/42 (38.1%)	0.011	-	-
Workplace exposure ≤ 15 yrs with small airways obstructive changes	4/33 (12.1%)			
Daily smokers with small airways obstructive changes	11/20 (55.0%)	0.002	18/68 (26.5%)	0.184
Daily smokers without small airways changes	9/55 (16.3%)		4/7 (57.1%)	
Passive smokers with small airways obstructive changes	5/20 (25.0%)	0.283	3/7 (42.9%)	0.134
Passive smokers without small airways obstructive changes	7/55 (12.7%)		8/48 (16.7%)	

Data are expressed as number and percentage of examinees with certain variable with and without obstructive pattern. Yrs: years.

* Tested by Chi-square test.

The effect of exposure duration in agriculture, smoking and age on lung functional parameters are shown in Table 9. Linear regression analysis showed that exposure duration, smoking and age had independent effect only on MEF_25-75_, and no effect on other functional parameters (FVC, FEV_1_, and FEV_1_/FVC).

**Table 9:** Effect of agricultural exposure duration, smoking and age on lung functional parameters.

	Beta	p
FVC		
Age	-0.158	0.476
Exposure duration	-0.093	0.653
Smoking (pack-years)	0.174	0.264
FEV_1_		
Age	-0.238	0.149
Exposure duration	-0.197	0.439
Smoking (pack-years)	0.298	0.426
FEV_1_/FVC		
Age	-0.198	0.457
Exposure duration	-0.232	0.256
Smoking (pack-years)	0.097	0.679
MEF_25-75_		
Age	-0.398	0.086
Exposure duration	-0.497	0.038*
Smoking (pack-years)	0.465	0.045*

FVC - force vital capacity; FEV_1_ - force expiratory volume in the first second; MEF_25-75_ – maximal expiratory flow at 25-75% of FVC; Level of statistical significance:

* P<0.05; Tested by Multiple Linear Regression Analysis.

## Discussion

Chronic respiratory symptoms, lung functional impairment and respiratory disorders are currently important clinical and public health issues for agricultural workers worldwide. Numerous studies in this domain conducted within last few decades have proven a significantly increased risk of respiratory morbidity and mortality among farmers, documenting the relationship between occupational exposure to respiratory hazards in agriculture and occurrence of chronic respiratory symptoms, which results in further on development of chronic lung diseases [3].

The actual study compared the prevalence of chronic respiratory symptoms and lung function parameters, and further examined their relation to duration of exposure and smoking between agricultural and office workers.

Our previous studies showed that specific occupational exposure in agricultural workers can provoke certain respiratory health impairments, which are generally preventable, and closely related to its duration, characteristics, and intensity [28, 29].

A higher prevalence of chronic respiratory symptoms and lung function impairment in agricultural workers compared to other occupations was proven by an extensive amount of epidemiological and clinical studies in the field. The frequency of respiratory symptoms is closely related to the main type of agricultural activity, and mostly depends on intensity and duration of organic dust exposure. Some studies showed lower frequency of respiratory symptoms in agricultural workers having grain and crop cultivation as main activity, compared to those involved in cattle breeding and livestock farming [30, 31].

The prevalence of overall chronic respiratory symptoms in our study was 29.3%. Cough was present in 20% of the subjects, while the rates of phlegm, dyspnea, wheezing, and chest tightness were 10.7%, 12%, 10.7% and 8%, respectively, being similar to the study of self-reported symptoms in European animal farmers [32]. Having this in mind, our actual study demonstrated a strong association between the agricultural exposure and development of respiratory symptoms.

When it comes to exposure duration, many farmers start working since adolescents, and frequently continue to work even beyond the age of 65 years [7], whereas concerning the smoking habit, compared to other occupational groups, the percentage of smokers is known to be a bit lower in farmers [33]. In our study, the frequency of daily smokers in agricultural workers was 26.7%. In our actual study the frequency of respiratory symptoms in the last 12 months was higher in agricultural workers with exposure more than 15 years than in those with less than 15 years of exposure, but reached significant difference only for overall respiratory symptoms and dyspnea.

The research conducted among animal farmers in North America, Europe and New Zealand [34, 35] noted an increase in work-related respiratory symptoms. It was shown that contact and work with horses was consistently associated with higher prevalence rates of chronic bronchitis, dyspnea, organic dust toxic syndrome and farmer’s lung, in comparison to other types of farming [35].

Lung function measured by spirometry often is reduced in farmers compared to controls. In the Turkish study focused on horse keepers, Tutluoglu et al. found sensitization to horse hair in 12.8% of grooms [36], obstructive ventilatory pattern was observed in 24.6% of them, 16% presented a FEV_1_/FVC ratio of less than 70%, and 28.6% showed restrictive ventilatory pattern. Heller et al. found a significant lower FEV_1_/FVC ratio in subjects, daily exposed to dairy cattle and silage, compared to other farmers and controls [37]. Dosman et al. found a lower FEV_1_ and FVC in swine farmers compared to controls, although with a modest increase in FEV_1_/FVC ratio among swine farmers, suggesting presence of a mixed lung functional impairment [38]. In this context, a Canadian study showed a significant lower FEV_1_/FVC among swine confinement workers than in controls [39].

Recent studies demonstrated that the increased annual decline in lung function is usually associated with occupational and environmental exposures, such as smoking, dust, disinfectants, automatic dry feeding systems and endotoxin [5]. Our study confirmed the decline of lung functional parameters with the increase of exposure duration in agriculture, but statistical significance was reached only for MEF parameters in workers exposed more than 15 years, compared to those with less than 15 years of agricultural exposure.

The Canadian study suggested that there is a positive interactive effect of grain farming exposure and smoking on lung function and the prevalence of chronic bronchitis in women [31]. Our study showed that association between obstructive pattern and daily smoking, smoking experience, and pack-years smoked was not significant in exposed current smokers, as well as a joint effect of duration of exposure, current smoking, smoking experience, and pack-years smoked on obstructive pattern development in agricultural workers. We have found significant association between small airways obstructive changes in exposed daily smokers and smoking experience, as well as between small airways obstructive changes and exposure duration and pack-years smoked. The joint effect of exposure duration, daily smoking, smoking experience and pack-years smoked on small airways obstructive changes in agricultural workers was also significant, which was not the case for small airways obstructive changes with passive smoking.

Concerning the animal contact, Mazan et al. found that exposure to the equine barn environment for 10 h/week is a significant predictor of self-reporting respiratory symptoms within the past 12 months [40].

Possible differences in the frequencies of chronic respiratory symptoms and lung diseases may be in correlation with different age ranges of the included populations, or determined by heterogeneity of the occupational exposures [41]. By the cross-sectional study of Danish farmers, Iversen found a prevalence of 27% for chronic bronchitis and 8% for bronchial asthma [42], whereas a lower prevalence of asthma (5.3%) was found by Dalphin et al. among French farmers [43]. When compared to other non-farming occupations from the same region, farmers usually present higher prevalence of respiratory symptoms, which may not always be evident [44], because symptomatic farm workers may leave farming more often than asymptomatic ones, bringing on the surface the “healthy worker effect”. Usually, farming has been associated with respiratory symptoms in workers exposed to livestock [45, 46], compared to those involved in crop production has been much less studied.

Iversen et al. in a Danish study with a 5-year follow-up, reported that he annual decline in FEV_1_ was highest in pig farmers, followed by farmers with both pig and dairy production, and lowest in farmers with no animal production [47]. In another 7-year follow-up study from the same group, the annual decline in FEV_1_ was greater among swine farmers compared to dairy farmers, while in non-smokers the increased annual decline in swine farmers was 17 mL, compared to dairy farmers [48]. The Croatian study showed that the prevalence of chronic symptoms among male farm workers was greater compared to male control subjects, being significant for chronic cough, chronic phlegm, and chronic bronchitis, whereas among women farm workers, a significant difference was noted for chest tightness. Concerning ventilatory capacity tests in male farm workers significant coefficients were demonstrated for employment and smoking [49]. Our study confirmed that exposure duration, smoking, and age had independent effect only on MEF_25-75_, and no effect on other functional parameters (FVC, FEV_1_, and FEV_1_/FVC).

The present study has some limitations. First of all, relatively small number of the subjects in the study groups may have certain implications on the obtained results. Also, the absence of skin prick testing to common and workplace allergens could aggravate clear relationship between allergen sensitization and respiratory symptoms, as well as lung function parameters.

In conclusion, the actual study found higher prevalence of respiratory symptoms in the last 12 months with significant difference for cough and dyspnea, as well as significantly lower values MEF parameters in agricultural workers than in controls. Development of chronic respiratory symptoms was closely related to agricultural exposure duration and daily smoking, while pulmonary functions of agricultural workers have been found to be decreased related to the exposure duration and daily smoking, but reached significance only for small airways changes. Exposure duration, smoking and age had independent effect only on small airways changes.

The obtained results recognized the role of occupational exposure in agricultural workers in development of respiratory impairment, but also confirmed interactive influence of agricultural workplace exposure and daily smoking on development of chronic respiratory symptoms and airflow limitation, primarily targeting the smaller airways. Therefore, preventive measures should be focused on smoking cessation and effective tobacco control measures, in order to prevent the interaction and joint effect of smoking and workplace environment.
